# A Reduction in a Drug Account

**Published:** 1915-07-03

**Authors:** 


*To the Editor of* The Hospital.

### A Reduction in a Drug Account.

*To the Editor of* The Hospital.

Sir,—I note with much pleasure your report in The Hospital of June 26 *re* the drug account of the Surrey Dispensary. You also remark that it might be helpful to know by what means this is brought about.

On this I hope you will allow me to enlighten you. I respectfully put this to the credit of the dispenser, Mr. A. J. Hurn, M.P.S., who has held this appointment for twenty-six years, and known to me during this period. He attributes the reduction in the drug account to the fact of his preparing several concoctions, infusions, etc.

Further, he had the support of the medical staff to reduce the "German" preparations, of which a large quantity had previously been prescribed.

I feel confident that you will agree with me, and trust you will see this in the same light as myself. Pray pardon me for trespassing upon your valuable time and paper should you be pleased to acknowledge this in your next issue. This would please and oblige us all round, and be greatly esteemed by the writer.—With respectful compliments, faithfully yours, W. J. B.
June 28, 1915.

[Our correspondent's suggestion appears to be that when German preparations ceased to be prescribed the drug bill was diminished. Could not, then, the successful exertions of the dispenser and the staff have been employed before?—Ed. The Hospital.]

